# Feasibility of a Combined Aerobic and Strength Training Program and Its Effects on Cognitive and Physical Function in Institutionalized Dementia Patients. A Pilot Study

**DOI:** 10.1371/journal.pone.0097577

**Published:** 2014-05-20

**Authors:** Willem J. R. Bossers, Erik J. A. Scherder, Froukje Boersma, Tibor Hortobágyi, Lucas H. V. van der Woude, Marieke J. G. van Heuvelen

**Affiliations:** 1 University of Groningen, University Medical Center Groningen, Center for Human Movement Sciences, Groningen, The Netherlands; 2 VU University, Department of Clinical Neuropsychology, Amsterdam, The Netherlands; 3 University of Groningen, University Medical Center Groningen, Department of General Practice, Elderly Care Medicine, Groningen, The Netherlands; 4 University of Groningen, University Medical Center Groningen, Center for Rehabilitation, Groningen, The Netherlands; University of Glasgow, United Kingdom

## Abstract

**Objectives:**

We examined the feasibility of a combined aerobic and strength training program in institutionalized dementia patients and studied the effects on cognitive and physical function.

**Methods:**

Thirty-three patients with dementia, recruited from one nursing home, participated in this non-randomized pilot study (25 women; age = 85.2±4.9 years; Mini Mental State Examination = 16.8±4.0). In phase 1 of the study, seventeen patients in the Exercise group (EG) received a combined aerobic and strength training program for six weeks, five times per week, 30 minutes per session, in an individually supervised format and successfully concluded the pre and posttests. In phase 2 of the study, sixteen patients in the Social group (SG) received social visits at the same frequency, duration, and format and successfully concluded the pre and posttests.

**Results:**

Indices of feasibility showed that the recruitment and adherence rate, respectively were 46.2% and 86.3%. All EG patients completed the exercise program according to protocol without adverse events. After the six-week program, no significant differences on cognitive function tests were found between the EG and SG. There was a moderate effect size in favor for the EG for the Visual Memory Span Forward; a visual attention test. There were significant differences between groups in favor for the EG with moderate to large effects for the physical tests Walking Speed (p = .003), Six-Minute Walk Test (p = .031), and isometric quadriceps strength (p = .012).

**Conclusions:**

The present pilot study showed that it is feasible to conduct a combined aerobic and strength training program in institutionalized patients with dementia. The selective cognitive visual attention improvements and more robust changes in motor function in favor of EG vs. SG could serve as a basis for large randomized clinical trials.

**Trial Registration:**

trialregister.nl 1230

## Introduction

Cognitive and physical function declines with dementia and the decline accelerates with disease severity. Unsurprisingly, disease severity is the most reliable predictor of institutionalization and care burden [1], [2]. It is suggested that physical activity is a treatment modality which may have positive effects on dementia patients' cognitive and physical function and therefore could favorably influence the disease progression in institutionalized patients with dementia [3], [4]. However, previous exercise studies with dementia patients focused mainly on aerobic-only training and the results are inconsistent and less convincing compared with studies that included healthy older subjects [5]. In those studies with healthy older subjects a combination of aerobic and strength training was most effective in improving memory and executive function (e.g. inhibition, planning, impulse control, set-shifting) [6], [7]. Therefore, the emerging hypothesis is that a combined aerobic and strength exercise program for dementia patients may also be effective to improve memory and executive function.

Beneficial effects of a combined aerobic and strength training program on cognitive function may emanate from the complementariness between the neurobiological and physiological mechanisms underlying the individual exercise programs. Aerobic and strength training each can positively affect the levels of insulin like growth factor-1 and brain derived neurotrophine factors [7], [8]. These proteins mediate neuronal cell growth, proliferation, survival, and differentiation. However, strength training may specifically lower the levels of the neurotoxic homocysteine, which is related to improved cognition [7], [9]. Furthermore, strength training improves critical elements of physical function, such as mobility and balance [10], [11]. The strength training effect could in turn allow patients to perform the aerobic exercise component at a higher intensity. Aerobic training at a moderate to high intensity level can improve cerebral blood flow [12], a factor known to mediate effects on cognitive function [13]. Altogether, there is a reasonably strong potential that a combination of aerobic and strength training could improve cognitive and physical function in patients with dementia.

A combined program of aerobic and strength activities was found feasible and effective to improve global cognitive and motor function in community dwelling patients with mild dementia [14], [15]. In contrast to community dwelling patients, institutionalized patients with dementia are older and physically, cognitively, socially, and psychologically more vulnerable [2]. Therefore, an exercise program may be less feasible for this specific patient group. Although both aerobic and strength training studies with institutionalized dementia patients are available, there is no data available of how feasibility is affected by a complexity of alternating strength training with aerobic training. This complexity is important because dementia patients may perceive variation or change in activities and environment as upsetting or disruptive in daily-life routine, causing stress and leading to not attending to activities, such as exercise [16]. Last, the session-frequency in previous studies ranged between 1–7 sessions per week [5] but no extensive data on adherence rates was reported in these studies. Therefore, it remains unclear at what frequency institutionalized dementia patients are able and/or willing to exercise, which is important because the American College of Sports Medicine (ACSM) guidelines recommend 3–5 times weekly exercise at a moderate to high intensity level, with alternating aerobic and strength training, to improve physical fitness [17]. All in all, prior to investigate any potential effects of high frequency combined exercise in a frail institutionalized moderate-severe dementia population, it is required to first study the feasibility of such a training program (e.g. 5 sessions per week, alternating aerobic with strength training). Here we define an intervention feasible when adherence to the program is high (>75%), the number of drop-outs is low (<20%), there are no adverse events, and participants complete the program according to protocol.

Taken together, the purpose of the study was to determine the feasibility of a combined aerobic and strength training program and to perform an exploratory analysis concerning the effects of such an exercise program on cognitive and physical function in institutionalized patients with dementia.

## Methods

### Ethics statement

The Medical Ethics Committee of VU Amsterdam, The Netherlands approved the research protocol according to the principles expressed by the Declaration of Helsinki. This clinical trial is registered at the Netherlands Trial Register (trial number 1230). The protocol for this trial and supporting CONSORT checklist are available as supporting information; see Checklist S1 and Protocol S1.

### Study design and procedures

This is a non-randomized, two-group, pretest-posttest, and single-blind pilot study. Between March 2010 and October 2010, a geriatrician recruited all of the participants from a psychogeriatric ward in a nursing home in Haren, the Netherlands (N = 78). The inclusion criteria were as follows: 70 years or older, a diagnosis of dementia, not wheelchair bound, and able to walk independently ten meters with or without a walking aid. All participants and their relatives were fully informed about the details of the study, including the risks, time commitment, and the possibility to be assigned to a combined aerobic and strength exercise group (EG) or a social visits group (SG). Each patient's legal representative gave written consent.

**Figure 1:**
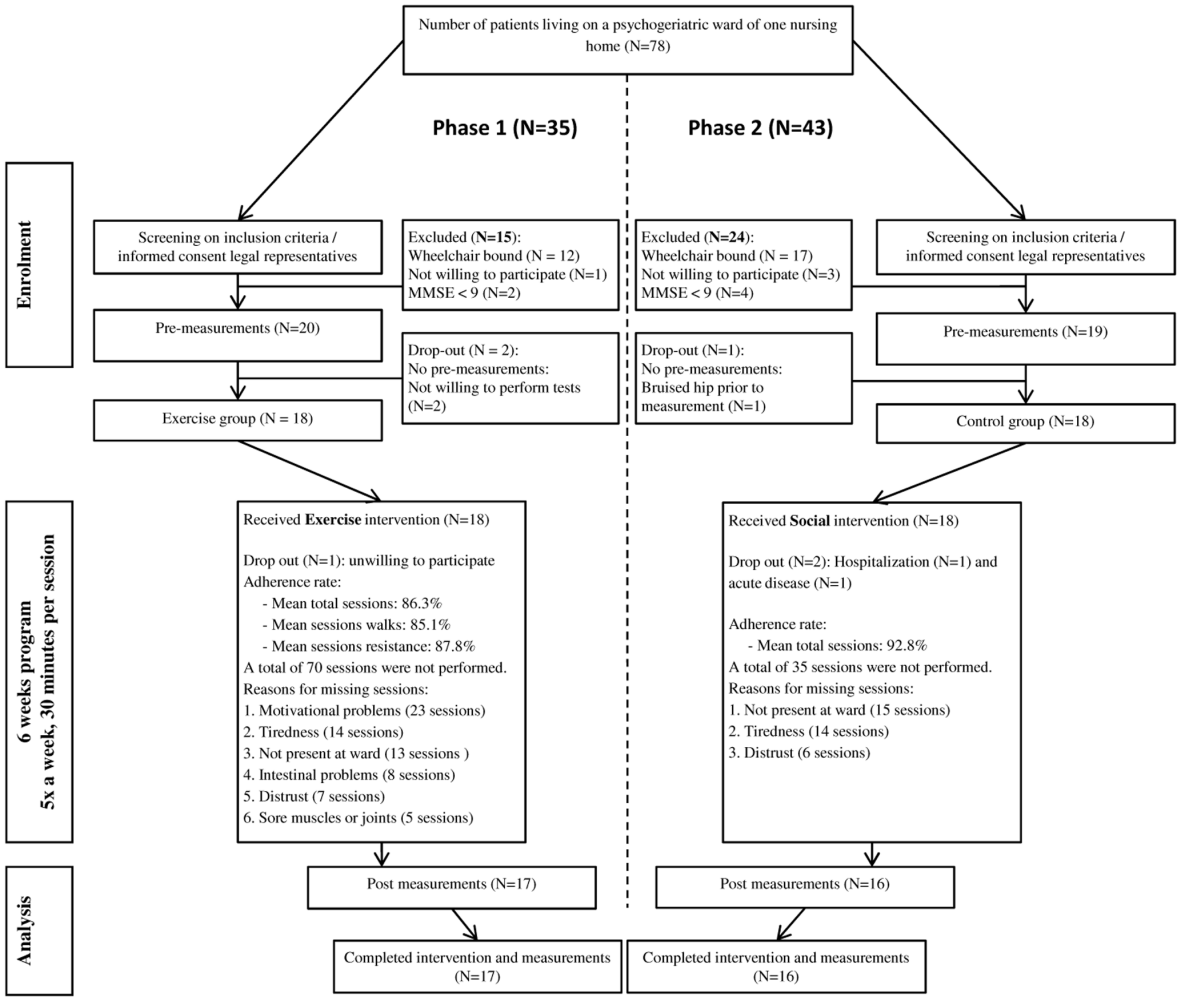
Flowchart of the study. Phase 1 (left side) determined feasibility by including a combined exercise group. Phase 2 (right side) explored the effects of the program by including a social visits group.

Because there are no previous studies on the feasibility of subjecting institutionalized patients with dementia residing in a psychogeriatric ward to a combined aerobic and strength training program, first we had to ascertain the feasibility that such a program can be executed at all in this specific setting with this specific population. Therefore, phase 1 of the study determined feasibility. The aim of phase 2 was to explore the effects of the program on cognitive and physical function and compare these effects to a social visits group. Figure 1 shows the flow of patient recruitment.

For phase 1 (March 2010–May, 2010), a geriatrician screened four out of eight wards in one nursing home where 35 residents with dementia were living, of whom 20 were enrolled in the study and 18 ultimately participated in the EG. Then, in phase 2 (August 2010–October, 2010), the same geriatrician screened the remaining other four wards (N = 43) from the same nursing home, of whom 19 were enrolled and 18 eventually participated in the SG.

The cognitive and physical assessments for all participants took place at baseline (T0) and after an intervention period of six weeks (T1). A trained research assistant, blinded for the intervention, administrated the cognitive and physical function tests at T0 and T1. The research assistant conducted the measurements at the same time of the day for a given patient.

### Intervention

Exercise and social visit sessions were provided in an individually supervised format for a period of six weeks. An individually supervised format was chosen because dementia is related to behavioral problems, such as apathy and agitation and this may cause inactivating thoughts and difficulties in initiating movement [1]. For safety reasons, institutionalized patients who live in a closed ward are required to have personal guidance for activities outside the ward. Each participant received five, 30-minute-long sessions during each of the six weeks. Four allocated trained research assistants delivered the two intervention programs and kept a log on the indices of feasibility.

#### Exercise group

Each week, the EG participated in three walking sessions and two strength training sessions. Walking sessions took place in the corridors of the nursing home or on paved outdoor walking paths near the nursing home. In accordance with ACSM guidelines for aerobic activity, our aim was to offer walking sessions at a moderate to high intensity level [17]. If a participant requested rest, an appropriate rest period was included in the 30-minute-long session. As soon as patients recovered, walking was resumed. The trainer monitored the intensity-level of the walks by asking the patients rate of perceived exertion (RPE). RPE was measured at the end of each session. The goal was to reach RPE-scores between 12–15 (e.g. ‘somewhat hard’ - ‘hard’, maximum score 20) [17].

In accordance with ACSM guidelines for strength training, the EG participated in two strength training sessions per week. These sessions were conducted in the patient's personal room on two non-consecutive days with at least 48 h between sessions [17]. Strength exercises focused on lower-limb strengthening to activate large muscle groups known to contribute to gait speed, balance, and mobility [11], [18]. The exercises were as follows: (1) seated knee extension, (2) plantar flexion through toe raises, while holding both hands of the trainer, (3) hip abduction by moving the straight leg sideways, while standing behind and holding on to a chair, and (4) hip extension by moving the straight leg backwards, while standing behind and holding on to a chair. An exercise program targeting the same muscle groups was successful in improving mobility and leg strength in community dwelling older adults, recovering from a hip fracture [19]. Exercise intensity increased gradually by increasing the number of repetitions and by affixing small weights to the ankle. In accordance with ACSM guidelines for strength training, our aim was to offer a moderate to hard intensity strength exercise program at an RPE of 12–15 (e.g. ‘somewhat hard’ - ‘hard’) [17]. To minimize the chance for an injury, overload, and drop-out, all participants started with three sets of 8 repetitions for each leg without weights. When an exercise was performed with ease and according to protocol as judged by the trainer (RPE<12 e.g. fairly light), the number of repetitions was increased to 10 in the next session, and 12 thereafter. When a participant was able to correctly perform 12 repetitions without weights, at an RPE<12, 0.5 kg was attached to the ankle. After the weight was attached, patients performed eight repetitions and progressed as described before. The trainers increased the weights from 0 kg to a maximum of 1.5 kg in 0.5 kg increments to reach RPE 12–15. For plantar flexion, the number of repetitions increased in increments of 2 with each session, to a maximum of 30 repetitions.

#### Social group

The SG received individualized social visits for the same duration and frequency as the EG. Social engagement may have a positive effect on cognitive function in patients with dementia [20]. Therefore, to control for social engagement, activities during social visits were small talk while sitting in a chair.

### Measurements

#### Feasibility

We obtained the recruitment rate at the psychogeriatric ward of one nursing home for participating in the combined exercise program. We defined an intervention feasible when the rate of program adherence is high (>75%), the number of drop-out low (<20%), no adverse events occur, and participants complete the program according to protocol.

#### Cognitive function

Cognitive function was measured with a neuropsychological test battery that covered the domains global cognitive function, verbal memory, visual attention, and executive function. Tests used in this study were based on a review [21] and a study-protocol [22].

Mini Mental State Examination (MMSE) measures the global level of cognitive function [23]. Scores range from 0–30.

Faces Recognition Test, as a subtest of the Rivermead Behavioral Memory Test (RBMT), measures visual long term memory/attention [24]. Version A was used. Five cards with faces were shown to the participant, each for five seconds. After ten minutes, ten cards were presented; five cards were shown before and five cards presented new faces. The participant had to recognize if a face was shown before or not. The total score is the number of correct answers minus the number of incorrect answers. Scores ranged between −10 and +10. A higher score represented a better performance.

Pictures Recognition Test, as a subtest of the RBMT, measures visual long term memory/attention [24]. The versions A + B were combined. Ten cards with objects were presented, each card for five seconds. After ten minutes, twenty cards were presented; ten were shown before and ten cards presented new objects. The participant had to recognize if an object was shown before or not. The total score was the number of correct answers minus the number of incorrect answers. Scores ranged between −20 and +20. A higher score represented a better performance.

Visual Memory Span Forward and Backward, as a subtest of the Wechsler Memory Scale Revised (WMS-R), consisted of printed squares on a card [25]. Participants were instructed to repeat a number of tapping sequences that became longer with each trial. The test consisted of a forward and backward condition. The forward condition measured attention and immediate visual memory. The backward condition measured attention and visual working memory, an executive function. Scores of both sub-tests ranged between 0–14. A higher score represented better performance.

Digit Span Test Forward and Backward are subtests from the WMS-R [25]. The forward condition consists of increasingly long sequences of orally presented numbers at a rate of one digit per second. Participants were asked to repeat the exact same sequence of numbers immediately. In the backward condition participants were asked to repeat the sequence in reverse order. It is suggested that the forward condition taps more into the short-term memory and the backward condition more into working memory, an executive function. Each condition ends when a participant fails to recall two strings of the same length or repeated an eight-digit sequence correctly. The scores for both conditions range between 0–21.

8-Words Test Direct-Recall and Recognition is a list-learning test for people with memory problems [26]. Eight words were orally presented five times in a row. Every time, directly after presenting the words, participants were asked to repeat as many words as possible. The first outcome measure was the total number of correct words over five trials (Direct-Recall score 0–40). After 15 minutes the examiner orally presents 16 words among which eight words presented in the direct-recall phase and eight new words. The participant had to recognize if a word was presented before (Recognition score 0–16).

Verbal Fluency Test evaluated executive function [27], [28]. Participants were asked to name as many examples of ‘animals’ as possible in one minute. After a few minutes, they were asked to name as many examples of ‘professions’ in one minute. The outcome measure was the total number of animals and professions.

Picture Completion Test, as a sub-test of the Groningen Intelligence Test, measures visual perception and alertness to detail (executive function). During the test, incomplete drawn figures were presented [29]. Participants were instructed to describe the picture as a whole. The pictures increased in difficulty as the test progressed. With five false answers in a row the test was stopped. Scores ranged from 0–20, with one point for each correct answer.

Stroop Task consisted of three subtasks [30]. Each task was performed as quickly as possible over a timed period of 45 seconds. Card 1 presented black-printed words ‘red’, ‘green’, ‘blue’, and ‘yellow’. Participants were instructed to read as many words as possible in the right order (left to right, top to bottom). Card 2 presented solid colored patched blocks which were red, green, blue, and yellow. As many of these colors needed to be named in the right order. Finally, Card 3 presented colored words. That is, each word was printed in a different color (i.e. the word ‘blue’ printed in yellow ink). The ink-color needed to be named and the highly provocative word-reading response needed to be inhibited. The final score was Card 2 minus Card 3. A lower final score indicated a better performance of inhibition, an executive function.

#### Physical function

Physical function was measured with a test battery that covered the domains of walking endurance, lower limb strength, mobility, and balance as suggested previously [21], [22]. Participants were allowed to use a walking aid.

Six-Minute Walk Test measures walking endurance [31], [32]. Participants were instructed to walk as long a distance as they could in six minutes. Each pre-set round was 26.3 meters and the distance was measured until the nearest meter. Stops and rests were included in the test performance.

Sit-to-Stand Test measures lower body strength in older adults [33]. To increase feasibility, participants were allowed to use upper limbs to rise from the chair. Participants were asked to stand-up and sit-down as many times as possible in thirty seconds. Ending in a standing position was counted as 0.5 stand.

Quadriceps strength test measures strength of the quadriceps femoris muscle [34]. A participant was instructed to generate maximal isometric knee extension force of the left leg, then the right leg while resting the arms on the thighs. The test was performed three times with 30 seconds of rest between trials. During the measurements, the maximum knee extension force was not visible for the participant. The highest score was given in kilograms. At baseline the maximum knee extension force of both legs was recorded. The highest score of either the left or right leg was recorded as outcome score. Then, after the six-week-long intervention period, the strongest leg at baseline (e.g. left or right leg) was tested again for maximum knee extension force.

Timed Up & Go Test quantifies functional mobility [35]. Participants were asked to rise from a standard chair (0.47 m height, horizontal seat and armrests), walk three meter to an orange cone, walk around it, and return in their chair in fully seated position. The test was performed two times and the mean time in seconds was used as score.

Six-meter Timed Walk measures walking speed [36]. Participants were instructed to walk six meters at a comfortable pace in a straight line, actively passing a line set at six meters. The test was performed twice and the fastest time was noted. As an outcome measure, walking speed (meters per second) over the last five meters of the test was calculated to exclude start-up speed.

Frailty and Injuries Cooperative Studies of Intervention Techniques (FICSIT-4) measures static balance control [37], [38]. Participants were asked to perform four different stances: (1) feet together, (2) semi-tandem, (3) tandem, and (4) single-leg without assistive device. Every stance had to hold for 10 seconds. The score on the FICSIT-4 scale ranged from 0 to 5 with a higher score indicating better performance.

Figure of Eight Test measures dynamic balance [39], [40]. Participants walked two laps of a standard figure-eight trajectory, as quickly and accurately as possible. The fastest time of two trials was noted, and the results were converted into walking speed (meters per second). Furthermore, oversteps outside the Figure of Eight were noted.

### Statistical analysis

SPSS Statistics 20 was used for data management and analyses. Possible group differences at baseline between EG and SG were analyzed for personal characteristics as well as cognitive and physical outcomes using Mann-Whitney or Fisher's tests. Difference scores between posttest (T1) and pretest (T0) for the EG and SG were calculated followed by Mann-Whitney U tests to compare the difference scores between EG and SG. The two-sided level of significance for global cognitive function was set at p<.05. To correct for alpha-inflation we used Bonferroni corrected alpha values for the cognitive domains verbal memory (p<.05/3 tests = .017), visual attention (p<.05/3 tests = .017), and executive function (p<.05/5 tests = .010). The level of significance for the physical domain walking endurance was set at p<.05, lower limb strength at p = .025 (p<.05/2 tests), mobility at p = .025 (p<.05/2 tests), and balance at p = .012 (p <0.05/3 tests). To explore the relation between cognitive change and physical change in the total study population, we used Spearman's correlations between the difference scores of the cognitive and physical tests. The magnitude of effects between the EG and SG were displayed as a Cohen's *d* Effect Size (ES). ESs were calculated with Cohen's *d* formula: *d*  =  [(post _EG_ − pre _EG_) − (post _SG_ − pre _SG_)]/*Sqrt* [([*s* ^2^ pre _EG_ (n _EG_) + *s* ^2^ pre _SG_ (n _SG_)]/[n _EG_ + n _SG_]) + ([*s* ^2^ post _EG_ (n _EG_) + *s* ^2^ post _SG_ (n _SG_)]/[n _EG_ + n _SG_])/2] [41].

To adjust for changes in SG, which may be initiated by natural course or social engagement, the formula as stated above included both values for the EG and SG. Cohen's benchmarks were used to indicate small (*d* = 0.20), moderate (*d* = 0.50), and large (*d* = 0.80) ESs.

## Results

Table 1 shows that patients in the two groups at baseline were not significantly different in age, gender, use of a walking aid, and global cognitive function measured with the MMSE.

**Table 1:** Baseline characteristics of the Exercise group and the Social group.

Characteristic	Exercise group (N = 17)	Social group (N = 16)	p-value
Age, years (Mean ± SD)	86.1±3.8	84.1±5.7	.118^a^
Male (%)	23.5%	25.0%	.958^b^
Use of walking aid (%)	29.4%	37.5%	.709^b^
Mini Mental State Examination (Mean ± SD)	16.5±4.4	16.8±4.3	.822^a^

Note: ^a^, Mann-Whitney U Test, ^b^, Fisher's Exact Test: p<.05.

### Feasibility

We managed to recruit 33 (46%) of 78 patients in a Dutch nursing home. Figure 1 shows that the rates of adherence (EG = 86%/SG = 93%) were similar in the two groups and it also shows reasons for non-participation such as motivational problems and tiredness. Three dropouts occurred (e.g. 2 due to not willing to perform the pretests, 1 due to injury) but these dropouts were not related to the intervention. Adverse effects included sore leg muscles (6 participants EG; 0 SG) and a sense of exertion (12 participants EG; 0 SG) but resulted in no dropouts. In total, all of the participants in the EG completed the 30-minute walks according to protocol. Further, 15 of 17 participants completed all strength exercises according to protocol. Two participants were unable to perform the toe rises while standing with the trainer's assistance and these patients performed toe rises in the seated position when lifting the heels.

### Changes in cognitive function

Table 2 presents the cognitive function data. The two intervention groups did not significantly differ at baseline in all cognitive tests. There were no changes in MMSE after either interventions (EG: 0%, SG: −3%). Performance in Faces Recognition Test, Pictures Recognition Test, and Visual Memory Span Forward was 6%, 22%, and 15% higher, respectively, in the EG, while the corresponding values in the SG were 2%, 13%, and 6% lower (*d* = 0.13, *d* = 0.46, *d* = 0.68, respectively). There were no differences between the EG and SG in the verbal memory tests. Finally, the EG scored 12%, 19%, and 13% higher on the Digit Span Backward Test, GIT incomplete figures test, and STROOP test, respectively. In comparison, the SG scored 8%, 8%, and 6% higher on these tests, respectively (*d* = 0.21, *d* = 0.27, *d* = 0.23, respectively). None of the between-group differences in cognitive test scores reached statistical significance.

**Table 2:** Cognitive function data for the Exercise group and Social group at baseline (T0) and after 6 weeks (T1).

	Exercise group				Social group				Exercise vs. Social		
Cognitive function tests	Pre-measure (T0) (Mean ± SD)	Post-measure (T1) (Mean ± SD)	T0 - T1 (%)	N	Pre-measure (T0) (Mean ± SD)	Post-measure (T1) (Mean ± SD)	T0 - T1 (%)	N	*d*	U	p*
*Domain: Global cognition*											
Mini Mental State Examination	16.5±4.4	16.5±4.6	0%	17	16.8±4.3	16.3±4.1	−3%	16	0.13	123.5	.657
*Domain: Visual attention*											
Faces Recognition Test	5.4±2.9	5.8±2.9	+6%	17	3.3±4.0	3.2±2.6	−2%	16	0.13	85.5	.114
Pictures Recognition Test	8.8±7.7	10.7±6.9	+22%	17	10.1±6.9	8.8±7.8	−13%	16	0.46^S^	90.0	.165
Visual Memory Span Forward	4.6±1.7	5.3±1.5	+15%	17	4.6±1.3	4.3±1.6	−6%	16	0.68^M^	67.5	.022
*Domain: Verbal memory*											
Digit Span Test Forward	8.9±3.2	9.8±2.1	+10%	17	8.4±2.8	8.9±2.4	+5%	16	0.17	118.5	.503
8-Words Test – Direct-Recall	14.5±7.3	15.1±6.7	+4%	17	12.8±8.1	13.2±7.4	+4%	16	0.01	126.0	.970
8-Words Test - Recognition	10.5±2.2	11.1±2.7	+5%	17	10.7±3.3	11.9±2.3	+12%	16	−0.27	126.0	.970
*Domain: Executive function*											
Verbal Fluency Test	13.6±8.1	13.9±7.7	+2%	17	11.9±6.2	12.2±7.1	+3%	16	0.00	135.0	.986
Digit Span Test Backward	5.7±2.1	6.4±1.9	+12%	17	4.3±2.2	4.6±1.6	+8%	15	0.21^S^	112.5	.576
Picture Completion Test	4.9±2.3	5.9±2.0	+19%	17	3.9±2.4	4.2±2.8	+8%	16	0.27^S^	122	.631
Stroop Task	28.9±12.7	24.9±12.5	+13%	15	23.8±11.8	22.4±8.6	+6%	11	0.23^S^	75.5	.721
Visual Memory Span Backward	3.7±2.0	3.9±1.5	+5%	17	2.8±1.8	2.9±1.4	+4%	16	0.03	109.0	.502

Note: SD, Standard deviation; T0, Baseline; T1, 6-week posttest;

S , small Cohen's *d* effect size;

M , medium Cohen's *d* effect size;

*, Bonferroni corrected p-values were set at p < 0.05 for the domain global cognitive function, p<.017 for domain visual attention, p<.017 for domain verbal memory, and p<.010 for domain executive function.

### Changes in physical function

Table 3 presents the physical function data. The EG and SG were similar at baseline in the physical function tests. Performance in the Six Minute Walk Test improved 17% in EG, and decreased 12% in SG (*d* = 0.70). Walking Speed also improved 18% in the EG but decreased 11% in the SG (*d* = 0.91). The EG improved 4% in knee extension strength while the SG lost 20% strength (*d* = 0.66). The EG gained 15% on the FICSIT-4 and the SG lost 1% (*d* = 0.43). No significant correlation larger than rho = .20 was found between any of the physical and cognitive change scores.

**Table 3:** Physical function data for the Exercise group and Social group at baseline (T0) and after 6 weeks (T1).

	Exercise group				Social group				Exercise vs. Social		
Physical function tests	Pre-measure (T0) (Mean ± SD)	Post-measure (T1) (Mean ± SD)	T0 - T1 (%)	N	Pre-measure (T0) (Mean ± SD)	Post-measure (T1) (Mean ± SD)	T0 - T1 (%)	N	*d*	U	p*
*Domain: Walking endurance*											
Six Minute Walk Test (m)	216.8±90.60	254.6±99.0	+17%	15	252.4±105.2	221.8±110.8	−12%	14	0.70^M^	51.00	.031*
*Domain: Leg strength*											
Sit to Stand test (no.)	7.9±3.3	8.7±3.3	+10%	16	8.8±3.0	8.3±3.2	−5%	16	0.41^S^	94.0	.210
Quadriceps strength test (kg)^a^	21.8±10.7	22.7±10.5	+4%	16	25.8±10.2	20.5±6.3	−20%	9	0.66^M^	28.0	.012*
*Domain: Mobility*											
Timed Up and Go Test (s)	18.5±8.7	16.6±6.2	+10%	16	17.6±7.4	18.4±8.9	+5%	16	0.35^S^	171.5	.102
Six-meter Timed Walk (m/s)	0.8±0.3	0.9±0.3	+18%	16	0.9±0.2	0.8±0.2	−11%	16	0.91^L^	51.0	.003*
*Domain: Balance*											
FICSIT-4	2.4±1.0	2.8±0.6	+15%	16	2.7±1.0	2.7±1.2	−1%	16	0.43^S^	92.0	.184
Figure of Eight speed (m/s)	0.4±0.2	0.5±0.2	+14%	16	0.5±0.3	0.5±0.2	−2%	16	0.32^S^	97.0	.254
Figure of Eight oversteps (no.)	6.7±6.2	5.8±6.6	+14%	16	5.9±8.1	7.8±9.3	−33%	16	0.39^S^	172.5	.093

Note: SD, Standard deviation; T0, Baseline; T1, 6-week posttest; FICSIT-4, Frailty and Injuries Cooperative Studies of Intervention Techniques;

S , small Cohen's *d* effect size;

M , medium Cohen's *d* effect size;

L , large Cohen's *d* effect size;

a , maximum knee extension force of the strongest leg (e.g. left or right) was measured at pretest and posttest; *, Bonferroni corrected p-values were set at p < 0.05 for domain walking endurance, p<.025 for domain leg strength, p<.025 for domain mobility, and p<.017 for domain balance.

## Discussion

The purpose of the study was to determine the feasibility of a combined aerobic and strength training program and to perform an exploratory analysis concerning the effects of such an exercise program on cognitive and physical function in institutionalized patients with dementia. Because this was a pilot study using small sample sizes, the results should be considered preliminary and viewed with caution. Still, the current results could serve as a basis for future larger randomized clinical trials.

### Feasibility

The individually supervised format of administering the intervention contributed to feasibility. We defined that an exercise program was feasible if the adherence rate was high (>75%), the number of drop outs low (<20%), no adverse events occurred, and the exercise program was performed according to protocol. We consider the mean adherence rate of 84% high and encouraging for future studies. Our rate was 31% higher than the mean adherence in a 12-month group-walking exercise program using patients with mild cognitive impairment [42] and 9% higher than a one-on-one 12-week home-based combined exercise program in a similar patient group [14]. The much shorter intervention period compared with the other studies could explain our substantially higher adherence rate. Therefore, a longer duration exercise program is needed to further study the adherence on a longer term.

All patients were able to perform the aerobic part of the training program according to protocol and 15 out of 17 patients performed the strength part of the training program according to protocol. A factor that contributed to the high program adherence was that all patients started the training program at a generally low-intensity, working their way up to higher intensity levels as the program progressed. The progressive training character, with a low baseline training intensity, may have contributed to higher adherence rates. As a result, no major adverse events other than sore leg muscles, a feeling of exertion, and no program related drop out occurred. Furthermore, progressive training is important because adherence rate in institutionalized patients with dementia is negatively affected by low self-efficacy and low external locus of control [43], [44]. Progressive, individually supervised training in the current study may have resulted in a more personalized approach and one-on-one feedback may have led to better participation during the exercise programs and higher adherence rates compared to group interventions. While an individually supervised format of treating institutionalized patients with dementia can be effective in keeping adherence rates high as in the present study, it is also time intensive and potentially costly. However, we experienced that perhaps the most effective way to deliver exercise programs in dementia patients is in an individually supervised format. This is supported by knowledge that patients with dementia suffer from behavioral problems, such as apathy and agitation, which may cause inactivating thoughts and problems in the initiation of movement [1]. A possible practical solution to offer a personal individualized training regime is to involve volunteers and family members to offer such programs in a structured and frequent way.

In total, 46% of the patients with dementia who lived in an institutionalized setting met the inclusion criteria and were willing to participate. Therefore, the generalizability of this study is limited to patients who are able to walk independently, with or without a walking aid and were willing to exercise. The main reason why walking and leg-strength training was not possible was that 37% of the residents was wheelchair bound. Different training strategies are needed for this group, which should focus at both aerobic training and upper-extremity training to maintain or improve handgrip and arm strength because these are key contributors to functional ability and independency in activities of daily life [45]. A possible exercise program could consist of an arm-bike exercise combined with upper body strength training.

### Cognitive function

A six-week exercise program did not significantly improve cognitive function. However, there was a medium effect size only for the Visual Memory Span Forward, a visuospatial attention and memory test. This finding is in line with other studies, demonstrating positive effects of exercise on visuospatial but not on verbal processes [46]. This selective effect on visuospatial memory was also found in a meta-analysis of Colcombe & Kramer [6] and suggested a mediating role for a specific part of the hippocampus. The involvement of the hippocampal area was shown by an aerobic exercise program, which led to increased anterior hippocampal volume, and had little effect on the posterior hippocampal part in healthy older adults [47]. Neurons found in the anterior part of the hippocampus were selectively associated with visuospatial memory [48], and are more prone to age related atrophy than the neurons in the posterior hippocampus [49]. So, the current study provides some evidence that these selective effects of exercise on visuospatial memory are also present in older adults with dementia who participated in our combined aerobic and strength training program. Again, these results need to be interpreted with caution and studied further in larger randomized clinical trials over a longer intervention period.

The present data failed to confirm previously reported improvements in executive function after an exercise intervention in frail sedentary cognitively-impaired older adults [4]. A possibility to achieve more change over time is to perform an exercise program over a longer intervention period (e.g. 12 weeks) [3], [4]. Furthermore, measurements in cognitively impaired older adults may be less sensitive to change compared with measurements in cognitively non-impaired older adults due to large score variation [50]. We attempted to control for possible intraday variation by performing the measurements at the same time of the day (e.g. morning or afternoon). However, a small sample size, and therefore the use of non-parametric tests, led to the use of difference scores, resulting in lower test reliability. Therefore, future studies should replicate this study with a larger sample size to eliminate this problem.

### Physical function

A combination of aerobic and strength exercise sessions led to improvements in tests that measured walking endurance, leg strength, and mobility. The current improvements of leg strength and functional mobility are also in line with results of a six-week, twice to three times per week strength exercise training program [51], emphasizing that a short-term six-week exercise program may already result in beneficial change of physical functions. These improvements should conceptually increase the ability to walk [10] and achieve higher VO_2_-peak levels during aerobic activities [52]. In turn, this may promote cerebral blood flow, which is associated with higher level of cognitive function [12], [53]. However, we found no association between physical function change scores and cognitive function change scores. We suggest this may relate to different rates of change over time between physical and cognitive function [54]. It remains to be seen if the motor improvements eventually result in correlated improvements in cognitive function in proportion to intervention duration or if the beneficial effects of exercise intervention reach a saturation point. Therefore, long-term intervention studies are needed.

## Conclusions

The present pilot study showed that it is feasible to conduct a high-frequency combined aerobic and strength training program in institutionalized patients with dementia. The data also show selective visual attention improvements in cognitive function and more robust changes in motor function in favor of the exercise compared with the social visit intervention. These initial positive results are encouraging and warrant the execution of randomized clinical trials involving higher patient numbers and longer duration.

## Supporting Information

Checklist S1 **CONSORT checklist.**
(DOC)

Protocol S1 **Trial study protocol 04209.**
(PDF)
