# Supplementary material for: Feasibility of a Combined Aerobic and Strength Training Program and Its Effects on Cognitive and Physical Function in Institutionalized Dementia Patients. A Pilot Study
Source: PLoS One. 2014 May 20;9(5):e97577. doi: 10.1371/journal.pone.0097577 (PMC4028210; doi:10.1371/journal.pone.0097577)
Supplement: Protocol S1 — Trial study protocol 04209. (PDF) [file pone.0097577.s002.pdf]

Research Protocol

The effects of physical activity on cognition and behaviour in older adults with Alzheimer's Disease

Drs. Laura Eggermont & Prof. dr. Erik Scherder
Vu University, Amsterdam
Department of Clinical Neuropsychology
Van der Boechorststraat 1, 1081 BT Amsterdam,
LHP.Eggermont@psy.vu.nl
July, 2005

SUMMARY

Title: The effects of physical activity on cognition and behaviour in older adults with Alzheimer's Disease

Rationale: Alzheimer's Disease presents a major public health problem that impacts people's ability to maintain cognitive, physical and social function. There are indications that physical activity can enhance cognition in older people with dementia. However, the number of studies is limited, the outcomes ambiguous and only studies with aerobic exercise programs were performed. This study focuses on the feasibility and effects of combined strength and aerobic exercise in older people with dementia.

Objective: The objective is to investigate the feasibility and effects exercise on cognition, behavior and physical functioning.

Study design: This is a non-randomized, two-group, pretest-posttest, and single-blind design. A geriatrician is involved in the recruitment of all of the participants who live in a psychogeriatric ward in a nursing home. Participants will be assigned to an exercise group or a control group. Each patient's legal representative gave written consent. Measurements will take place, blinded for group, before the intervention (pretest) and after the 6-week intervention (posttest)

Study population: The study population consists of older people with moderate to moderate severe Alzheimer's Disease (MMSE 9-23) aged > 70 years of age.

Intervention: The intervention consists of a supervised physical exercise program which will be offered for 30 minutes a day, five days a week, during 6 weeks. The controls receive social visits with the same frequency and duration.

Main study parameters/endpoints: The main outcome parameters are feasibility, cognition, behaviour, and physical functioning.

Nature and extent of the burden and risks associated with participation, benefit and group relatedness: Burdens for the participants are the exercise program, as well as the physical test battery and neuropsychological test battery. The total test battery takes approximately 65 minutes per session for each participant. Exercises and tests are safe and feasible for frail older people with Alzheimer's Disease. All activities in this study are within the range of normal activities of daily life. Participants are provided with individual supervision, they stay in their own familiar environment and specialized healthcare personnel are always nearby. The project group believes that research to investigate the effects of the intervention and the measurements described in this protocol brings no increased risks to the subject.

INTRODUCTION AND RATIONALE

The incidence of Alzheimer's Disease or more general dementia, increases with age and it presents a major public health problem that impacts people's ability to maintain occupational and social function (Lui-Ambrose & Donaldson 2009). As the world population ages, the number of people suffering from dementia will increase. In 2006, there were 24 million people with dementia worldwide, a number that will increase to 40 million people in 2020 and to 80 million in 2040 (Ferri et al., 2005). In 2005, the Netherlands spend almost 5% (3,2 billion euro) of all healthcare costs on dementia healthcare. Strategies that would prevent the progression of cognitive impairment would have enormous economical and societal value.

Dementia is characterized by cognitive decline and subsequently behavioral problems and limitations in the performance of activities of daily living (ADLs). In addition, physical functioning is often affected by dementia. Finally, dementia leads to loss of independence and institutionalization. Therefore, for people with dementia "Healthy Aging" means to slow down the cognitive and physical decline as much as possible. Treatments to prevent or to reduce the consequences of dementia are extremely important. The most prevalent treatment is the prescription of medications to affect the cognitive and behavioral problems. Pharmacological treatments, however, have limited effects and may have unacceptable side effects (Salloway et al., 2009). Therefore, there is an urgent need for non-pharmacological treatments. Physical exercise may be such a treatment.

Kramer et al. (1999) investigated whether greater aerobic fitness in cognitively non-impaired older adults would result in selective improvements of executive control processes. 124 sedentary older adults were randomly assigned to either aerobic (walking) or non-aerobic (stretching and toning) exercise. Those who received aerobic training showed substantial improvements in cognitive performance on tasks requiring executive control compared with non-aerobically trained subjects. Further, in a recent review study of Liu-Ambrose (2009) evidence is provided that strength training also has cognitive benefits for cognitively non-impaired older people. Finally, a combination of aerobic and strength training may lead to even better results for cognitive functioning in cognitively non-impaired older people (Kramer & Colombe, 2003).

For people with dementia, the effects of physical activity on cognition are less clear. Studies are scarce, and with ambiguous results (Scherder et al., 2007). Furthermore, these studies focused only on the effects of aerobic exercise (mostly walking programs) (Scherder et al., 2007; Lui-Ambrose et al., 2009). The studies involving older people with dementia vary with respect to stage of dementia, number of participants, frequency, duration of the intervention and intensity. In some studies with dementia patients, a positive effect was found on cognition after 10–24 weeks of aerobic exercise

(Lautenschlager et al., 2008; Friedman et al., 1991; Burns et al., 2008; Palleschi, 1996). In contrast, other studies did not find an effect on cognition after 10-16 weeks of aerobic exercise (Sobel et al., 2001; Cott et al., 2002).

Because strength training can influence cognition in cognitively non-impaired older people, strength training may also be of importance to influence cognition in older people with dementia. Muscle weakness, may be caused by neuromuscular weakness, and is a prominent symptom among people with central nervous system disorders, such as dementia (Thomas et al., 2003). People with dementia have a number of symptoms that might primarily reflect muscular dysfunction, likely through changes in the recruitment and activation of motor units or a decrease in muscle fiber contractile properties. As a consequence, lower-extremity strength is affected and basic activities such as walking and transferring, as well as the maintenance of balance are disturbed (Lexell et al., 2000). This could lead to a decreased aerobic intensity level during walking, resulting in less or no improvement in aerobic capacity. Also, leg strength correlates directly to step length and walking-speed, which is important for improving physical functioning and thereby, improvement in VO2max (Scarborough et al., 1999). Hence, in people with dementia, muscle strength may be a necessary condition to improve aerobic capacity. Also, Thomas et al. (2003) already showed that it is feasible and physically effective to include lower extremity strength training in older people with dementia. Thus, previous studies suggest including strength training in exercise programs for older people with dementia to improve physical and cognitive functioning.

In conclusion, the effects on cognition, physical functioning and ADL of a combined strength and aerobic exercise intervention have never been investigated in older people with dementia. There are potentially immense benefits to persons with dementia, their family caregivers, and the healthcare system of managing or reducing the symptoms of dementia. Therefore, it is of great interest to gain knowledge about exercise as a non-pharmacological treatment.

1. OBJECTIVES

Primary Objective: The primary objective is to study the feasibility and effects of combined strength and aerobic exercise on cognition in order to improve physical activity programs for older people with dementia. There are three reasons to hypothesize that a combination of strength training and aerobic exercise may influence cognition stronger than solely aerobic exercise. First, strength training may have an independent effect on cognition through reduction of serum homocysteine and increase in insulin growth factor-1 (IGF-1) concentrations (Liu-Ambrose, 2009). Increased homocysteine concentrations are associated with impaired cognitive performance (Schafer et al., 2005), IGF-1 promotes neuronal growth, survival and differentiation and thus improves cognitive performance (Cotman et al., 2002). Second, older people with dementia often have reduced muscle strength and mobility (Scherder et al., 2007). This limits the intensity of aerobic training and subsequently hampers the chain of reactions that lead to the improvement of cognitive functioning. Even in older people with dementia, strength training appeared to be effective in improving muscle strength and mobility (Thomas et al., 2003). Consequently, aerobic exercise can be performed with a higher intensity which may lead to stronger effects on cognition. Third, in people without dementia, combined strength and aerobic exercise appeared to affect cognition stronger than aerobic exercise alone (Colombe & Kramer, 2003). Furthermore, we investigate the effects of combined strength and aerobic training on physical functioning and ADLs of older people with dementia because physical functioning may mediate the effects of physical activity on cognition (Kramer & Columbe, 2003).

Figure 1 shows a flow chart of the recruitment and intervention phase of the study.

When the informed consent is received by the researcher, a Mini Mental State Examination (MMSE) and a Timed Up & Go test for mobility will be administered by a trained student. If the participant scores between 10 and 23 on the MMSE and is able to perform the Timed Up & Go, he/she is included in the study. The administration of the tests and the execution of the intervention will be performed by trained master students Human Movement Sciences.

Total duration of the study is 6 weeks. Assessment of the tests will take place, blinded for group, before the intervention (pretest) and after the 6 weeks intervention (posttest). Interventions and measurements take place on location, in a specialized nursing. Assessment of the neuropsychological and physical tests will be executed and supervised by research assistants.

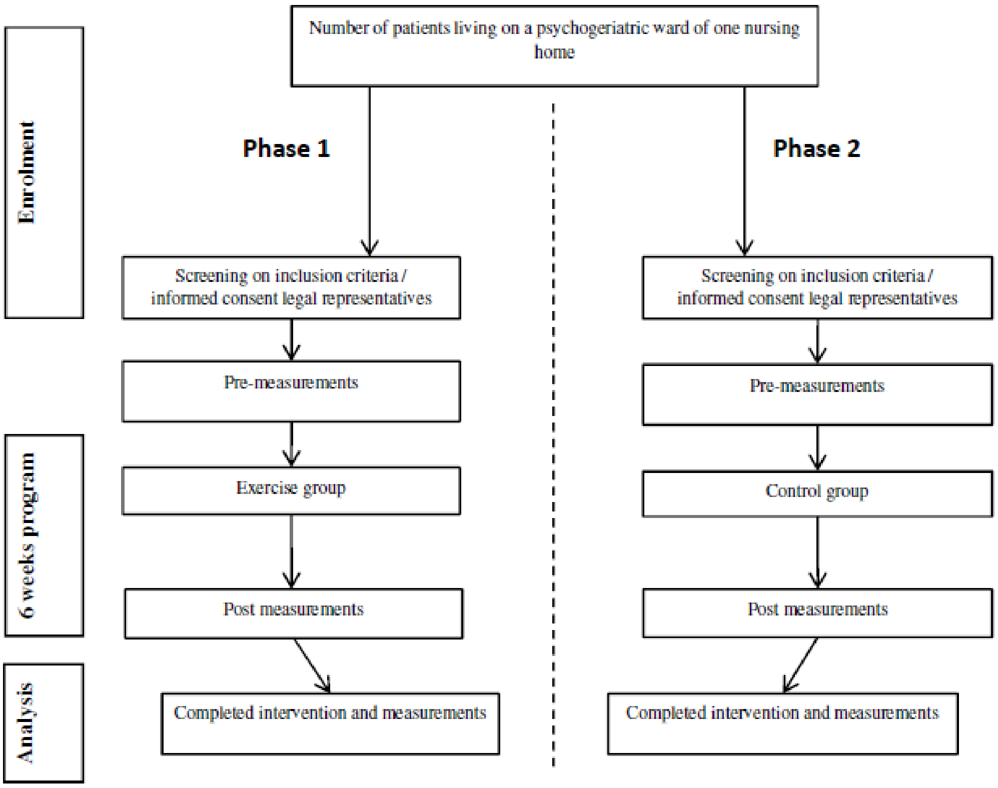

Figure 1. Flowchart of the recruitment and intervention phase of the study.

2. STUDY POPULATION

2.1 Population (base)

The research population consists of mobile older people >70 years of age and diagnosed according to their medical record with moderate to moderate severe dementia (MMSE 10-22) (Folstein et al., 1975). The participants will be recruited in specialized nursing homes in the north of the Netherlands.

2.2 Inclusion criteria

The geriatrician is informed about the activities of this study. According to the medical status, the geriatrician makes an inventory of potential subjects. Then, the geriatrician sends the information letter and informed consent to the legal representative. Subsequently, the legal representative can contact the investigator or the independent doctor for additional questions. The informed consent of the legal representative will be send to the investigator. If an informed consent is signed, the inclusion examination of the potential participant can start. A trained student will administer the tests for inclusion of the participant.

Criteria:

- To test if the participant is mobile to participate during the intervention and tests, the Timed Up & Go Test is assessed. The participant is included If the participant is able to perform this test with or without assistive device (Thomas et al., 2002).
- To test if the participant is able to perform neuropsychological tests and if he/she fits the population criteria, a MMSE is assessed. The participant is included if he/she scores between 9 and 23 on the MSSE (Folstein et al., 1975).

2.3 Exclusion criteria

The specialized nursing home doctor is informed about the activities of this study and the exclusion criteria. According to the medical status, the doctor makes an inventory of potential participants. The doctor excludes potential participants if they:

- are wheelchair bound
- have cardiovascular problems (e.a. severe high blood pressure or cardiac problems) that limit them from physical activity.
- have a history of alcoholism
- have severe visual problems
- have severe auditive problems

- have problems with the Dutch language

The geriatrician sends the information letter and informed consent to the legal representative. Subsequently, the legal representative can contact the investigator or the independent doctor for additional questions. The informed consent of the legal representative will be send to the investigator.

When the informed consent is received by the investigator, the investigator will assess a MMSE and Timed Up & Go test. If the potential participant sores < 9 or > 23 on the MMSE or is not able to perform the Timed Up & Go Test he/she is excluded form the study. The specialized nursing home doctor will contact the legal representative to inform about the exclusion of the participant in the study.

3. TREATMENT OF SUBJECTS

3.1 Investigational product/treatment

The supervised training programs will be offered for 30 minutes a day, 5 days a week, during 6 weeks (ACSM). The combined strength and aerobic group will walk 3 days per week and perform exercises to increase leg strength for 2 days per week. To control for social factors during the intervention, the control group will receive social visits with the same frequency and duration as the other group. For all training programs, the participants will be guided individually by the PhD student or a well-trained research assistant.

Combined aerobic and strength training

Aerobic training consists of walking. Walks will be performed for 30 minutes per session and, if necessary, moments of rest will be included. Walking will take place in the hallways of the specialized nursing home. If the participant is experiencing discomfort (e.a. pain, shortness of breath) the exercises are stopped. Assistance during walking or an assistive walking device is allowed. The aerobic training will be guided 1 on 1.In accordance with recommendation of the American College of Sports Medicine (ACSM), exercises to increase leg strength will be performed on nonconsecutive days with at least 48 hours between each session (ACSM 1998). The training will focus on lower-body strengthening. 30 minutes of specific strength exercises will follow: knee extensions (‘knee straightening’), plantar flexion ("toe standing"), hip abduction ("side lifts"), and hip extension ("back leg lifts"). These exercises are discussed with a geriatric physiotherapist and are derived from existing exercise programs used in hip revalidation and are being assessed in frail older people with high risk of falling. Hip revalidation showed that the exercises are feasible and therefore extremely suitable for older people with dementia. Participants will perform exercises that initially consist of 3 sets of 12 repetitions without weights. The intensity will be gradually increased using incremental weights (0.5; 1.0; 1.5 kg) which can be attached around the ankles and adding to the number of repetitions. These are standardized weights in revalidation which have proven to strengthen the lower extremities in frail older people (Thomas et al., 2002). During each exercise there will be paid attention to correct breathing and asking how the participant is feeling. Exercise sessions will take place in the living room where nursing home personnel are working. If the participant is experiencing discomfort (e.a. pain, shortness of breath) the exercises are stopped. The strength training will be guided 1 on 1.

Social visits

The control group will receive social visits with the same frequency and duration as the combined exercise group and the aerobic exercise group. Some examples of activities during the social visits

are reading the newspaper, or having a talk about daily things. Social visits will take place in the living rooms of the specialized nursing home. The social visits will be guided 1 on 1.

4. METHODS

4.1 Study parameters/endpoints

4.1.1 Main study parameter/endpoint

Feasibility and the differences between cognitive and physical test battery scores between pre and post measurement are the main outcome parameters for this study.

4.2 Study procedures

The neuropsychological measures and the physical measures are tasks specially designed for frail older people such as older people with dementia. The tests will take place on one day at 2 different parts of the day, thereby minimizing the burden for the participants. Tasks involved in the tests are responding to questions during the neuropsychological tests and performing activities of daily living such as walking or standing up from a chair. The neuropsychological tests will be guided 1 on 1. The physical tests for 1 participant will be guided by 2 trained Human Movement Science master students.

The study parameters consists of several outcome measures. These measures include global cognitive functioning, memory and executive functioning in older people with dementia, which are the domains to be expected to change in a positive way by the intervention. The neuropsychological tests are standardized test that are extensively described in the literature. The tests are commonly used and easy to administer. Coverage of verbal and non-verbal tests is taken into account (60% verbal, 40% non-verbal). All tests are feasible, reliable and valid for older people with dementia (Lezak 4th edition, 2004). The neuropsychological tests will approximately take 35 minutes and are administered three times (before the intervention (pretest), after the intervention (posttest) and at follow-up 10 weeks after the posttest).

Global cognitive functioning:
Mini Mental State Examination (MMSE) (Folstein et al., 1975)
The MMSE is a brief 30 item questionnaire test that is used to screen for cognitive impairment. It is used to estimate the severity of cognitive impairment at a given point in time and to follow the course of cognitive changes over time (Folstein et al., 1975). Therefore, the MMSE is an effective way to document an individual's global cognitive response to the intervention.

Memory:
Verbal Learning and Memory test (VLMT) (Lindeboom & Jonker, 1989)

The VLMT consists of the 8 Word Test (8-WT) and measures the short term and long term verbal episodic memory. A list of eight unrelated words is presented five times and after every time the participant has to recall as many words as possible. The direct recall score is the sum of words correctly mentioned in five trials. After 10 minutes, the delayed recall is assessed. The score is the number of correct words. To test ‘recognition’, a list of 16 words, including the eight words of the list, is read. The subject responds if the words were heard before. The score is the number of correct answers.

Digit Span Forward (DSf) (Wechsler, 1997)
The DSf measures the verbal short-term memory abilities. A series of verbally presented digits are asked to be repeated. The number of digits increases by one digit every three trials. The test is stopped with two consecutive errors. The score is the number of successful trials.

Visual Memory Span forward (VMSf) (Wechsler, 1955)
The VMSf is a test for short term memory. A series square blocks are pointed in a beforehand determined order. This order has to be repeated by the participant. The number of squares in each sequence increases by one every three trials. The test is stopped when the participant makes two consecutive errors.

Verbal Fluency Test (VFT) (Snijders & Verhage, 1983)
The VFT measures the ability to retrieve familiar information from the semantic memory. Also, executive functioning plays an important role during this task (Snijders, 1983; Wechsler, 1987). Participants have to say as many words as possible from a category in 60 seconds (1. animals; 2. professions). The number of animals and professions correctly produced in two separate sessions of 60 seconds is the score.

Executive functioning
Digit Span Backward (DSb) (Wechsler, 1955)
The DSb measures working memory, distractibility and attention/concentration, which are important in executive functioning. A series of digits have to be repeated in reverse order. The number of squares in each sequence increases by one digit every three trials. The test is stopped when the participant makes two consecutive errors.

Visual Memory Span backward (VMSb) (Wechsler, 1955)
The VMSb is a test for working memory and attention/concentration which are important subtasks of executive functioning. A series square blocks are pointed in a beforehand determined order. This order has to be repeated in reverse order by the participant. The number of digits in each

sequence increased by one digit every three trials. The test is stopped when the participant makes two consecutive errors.

Incomplete Figures (IF) of the Groningen Intelligentie Test (Snijders&Verhage, 1983)
The IF is a measure for abstract reasoning, which is a subtask of executive functioning. Silhouettes of incomplete images of objects and animals are shown and the participant has to deduce the meaning of the images. The test stops when the subject gives five consecutive wrong answers.

Rivermead Behavioural Memory pictures (RBMTp) (Wilson et al., 1985)
The RBMT picturers is a test for non-verbal long-term memory. The picture recognition test measures visual and verbal long-term memory. Executive functioning is highly involved in this task. 10 pictures of objects and animals are presented to the subject. After 5 minutes, the subject is asked to recognize the objects and animals, out of 20 pictures.

Rivermead Behavioural Memory Test faces (RBMTf) (Wilson et al., 1985)
The RBMT*f* is a test for non-verbal long-term memory. The faces recognition test measures visual long-term memory. Executive functioning is highly involved in this task. 5 faces are presented to the subject. After 5 minutes, the subject is asked to recognize the objects and animals, out of 10 pictures.

The order of the neuropsychological tests and a time indication in minutes:

1.	Mini Mental State Examination	10
2.	8-Word Test direct recall	5
3.	Incomplete Figures	2
4.	8-Word Test delayed recall / recognition	2
5.	RBMT faces recognition	1
6.	Digit Span forward / backward	3
7.	Verbal Fluency test animals	2
8.	Verbal Fluency test professions	2
9.	RBMT picture recognition	2
10.	Visual Memory Span forward / backward	5
	**Total:**	33 minutes

The physical tests used in this study are standardized, commonly used in studies with frail older people and are easy to administer. The tests will be administered in the specialized nursing homes and will take approximately 30 minutes. There are no extra risks for the participant because all test activities are a part of activities of daily living. Participants in this study are mobile older people

and are already familiar with the ADL tasks. Also, two students guide the participants during the tests to insure safety, minimizing the risk even further than in the normal situation. One participant is tested at a time. Between the tests the participant gets rest to recover from the test before. Several study parameters are assessed to measure the physical parameters leg strength, mobility, endurance capacity and balance.

5 times Sit-to-Stand test (5-STS) (Thomas et al., 2002)
The 5-STS is a physical performance test used to assess lower-extremity function and is the most commonly used tests in the literature for assessing leg strength in frail older people (Tiedeman et al., 2008). The time to perform the test is measured in seconds. The test will be stopped if the participant is not able to get out of a chair or takes longer than 60 seconds (Csuja et al., 1985).

Quadriso-tester (Verkerke et al., 2003)
The quadriso-tester is based on the system of Verkerke et al. (2003) and measures the strength in the musculus quadriceps femoris. The participant is instructed to sit in a chair with a force measuring device. The participant then builds up extension force with one leg. The test is performed three times per leg with 30 seconds of rest in-between. The highest score in Newton is registered in a computer program (QForce for Windows).

Timed Up & Go test (TUG) (Thomas et al., 2002)
The TUG measures mobility. The sitting participant stands up from a chair, walks 3 meters, makes a turn around a pylon, walks back and sits down in the chair. Participants are allowed to use their hands while standing up. Walking devices are allowed. The participants perform two trials. This test is feasible and safe for frail older people with mobility impairments (Podsiadlo et al., 2000). During the test a student walks with the participant to ensure safety.

6 meter walking test (6m-WT) (Steffen et al., 2002)
The 6m-WT is used to measure walking speed and step length (Guralnik et al., 1994). The subject walks a straight line. Subjects are allowed to use walking devices. Time is noted when the subject passed the finish line. The walked time is divided by the distance to derive gait velocity in m/s. During the test a student walks with the participant to ensure safety.

Six Minute Walk test (6MWT) (Tappen et al., 1997)
The 6MWT is an assessment for aerobic capacity (ATS, 2002). The participant is instructed to walk as far as possible in six minutes between two cones set 10 meters apart from each other. Walking devices are allowed and during the test the participant is allowed to take rests. The 6MWT is a reliability and feasible test in people with Alzheimer Disease and is preferred over the

2 minutes walking test (Tappen et al., 1997; Tiedeman et al., 2002). During the test a student walks with the participant to ensure safety.

Figure of Eight (FoE) (Johansson et al., 1991)
The FoE is administered to measure dynamic balance. Participants walk 2 laps of a standard 10-m figure-eight course, as quickly and accurate as possible. Walking devices are allowed. This is a reliable and feasible test for frail older people (Tegner et al., 1986). During the test a student walks with the participant to ensure safety.

FICSIT-4 (Rossiter-Fornoff et al., 1995)
The FICSIT-4 is assessed for static balance. During the FICSIT-4, the participants perform four different stances (feet together, semitandem, tandem, and single-leg) without assistive devices. Every stance has to hold for 10 seconds. During the test, 2 students stand at each side of the participant to ensure safety during the test. The FICSIT-4 is a test that is often used in studies with frail older people who have a high risk of falls.

The order of the physical tests and a time indication in minutes:

1.	6 meter walk test	3
2.	Figure of Eight	5
3.	5 times sit to stand test	2
4.	Timed Up & Go	2
5.	FICSIT-4	3
6.	Quadrisometer	5
7.	6 Minutes Walk test	10
	**Total:**	30 minutes

4.3 Withdrawal of individual subjects

Subjects can leave the study at any time for any reason if they wish to do so without any consequences. The investigator can decide to withdraw a subject from the study for urgent medical reasons.

4.3.1 Specific criteria for withdrawal

- The participant gets ill and can not participate in activities of the study.
- The participant gets transferred to another specialized nursing home.
- The participants resist with cooperation in the study.

4.4 Premature termination of the study

It is not likely that this study is prematurely terminated. If the study should be terminated in a premature stage, the problems at hand are tried to be solved, to continue the study in a later stadium. Problems at hand are discussed with the head of the department or healthcare personnel to be solved. Eventually, with approval of the discusser, the study will be resumed.

5. SAFETY REPORTING

5.1 Section 10 WMO event

In accordance to section 10, subsection 1, of the WMO, the investigator will inform the subjects and the reviewing accredited METC if anything occurs, on the basis of which it appears that the disadvantages of participation may be significantly greater than was foreseen in the research proposal. The study will be suspended pending further review by the accredited METC, except insofar as suspension would jeopardise the subjects' health. The investigator will take care that all subjects are kept informed.

5.2 Adverse and serious adverse events

For all participants, professional medical help is always present because the research takes place inside the specialized nursing homes. The professional medical help is always nearby and is responsible for the well-being of the participants. The investigators are responsible for monitoring the participants during activities and call in help if necessary. If an AE or SAE occurs, help of the specialized nurses is called in immediately and the protocol of the specialized nursing home is followed.

Adverse events are defined as any undesirable experience occurring to a subject during the study, whether or not considered related to [the investigational product / the experimental treatment. All adverse events reported spontaneously by the subject or observed by the investigator or his staff will be recorded.

A serious adverse event is any untoward medical occurrence or effect that at any dose:
- results in death;
- is life threatening (at the time of the event);
- requires hospitalisation or prolongation of existing inpatients' hospitalisation;
- results in persistent or significant disability or incapacity;
- is a congenital anomaly or birth defect;
- is a new event of the trial likely to affect the safety of the subjects, such as an unexpected outcome of an adverse reaction, lack of efficacy of an IMP used for the treatment of a life threatening disease, major safety finding from a newly completed animal study, etc.

All SAEs will be reported through the web portal *ToetsingOnline* to the accredited METC that approved the protocol, within 15 days after the sponsor has first knowledge of the serious adverse reactions.

SAEs that result in death or are life threatening should be reported expedited. The expedited reporting will occur not later than 7 days after the responsible investigator has first knowledge of the adverse reaction. This is for a preliminary report with another 8 days for completion of the report.

5.2.1 Suspected unexpected serious adverse reactions (SUSAR)

Not applicable.

5.2.2 Annual safety report

Not applicable.

5.3 Follow-up of adverse events

All adverse events will be followed until they have abated, or until a stable situation has been reached. Depending on the event, follow up may require additional tests or medical procedures as indicated, and/or referral to the general physician or a medical specialist.

6. STATISTICAL ANALYSIS

6.1 Descriptive statistics

Descriptive statistics will consist of measures of central tendency, median percentages and measures of variability (means, standard deviations, ranges) of subjects characteristics on pre-test, post-test and delayed post-test stratified by group.

6.2 Analysis

SPSS Statistics 20 is used for data management and analyses. Possible group differences at baseline between EG and SG are analysed for personal characteristics as well as cognitive and physical outcomes using Mann-Whitney or Fisher's tests. Difference scores between posttest and pretest for the exercise group and control group were calculated followed by Mann-Whitney U tests to compare the difference scores. To explore the relation between cognitive change and physical change in the total study population, Spearman's correlations are used between the difference scores of the cognitive and physical tests. The magnitude of effects between the EG and SG are displayed as a Cohen's d Effect Size (ES). ESs were calculated with Cohen's d formula:

d = [(post exp – pre exp) – (post cont – pre cont)] / Sqrt [([s2 pre exp (n exp) + s2 pre cont (n cont)] / [n exp + n cont]) + ([s2 post exp (n exp) + s2 post cont (n cont)] / [n exp + n cont]) / 2] [41].

For all statistical tests, a p-value of < 0.05 is used to assess statistical significance. Statistical analyses are conducted using SPSS version 20.0 (SPSS inc., Chicago, IL, USA).

7. ETHICAL CONSIDERATIONS

7.1 Regulation statement

The study will be conducted according to the principles of the Declaration of Helsinki (2008, Seoul) and in accordance with the Medical Research Involving Human Subjects Act (WMO).

7.2 Recruitment and consent

The geriatrician is informed about the activities of this study by the coordinating investigator. According to the medical status, the geriatrician makes an inventory of potential subjects. Then, the geriatrician contacts the legal representatives of these subjects and asks if they would like to receive information about the study. If the legal representatives are interested, information about the study and an informed consent is send to their address by the geriatrician. Subsequently, the legal representative can contact the investigator or the independent doctor for additional questions. The informed consent of the legal representative will be send to the investigator. The legal representatives have two weeks to consider participating in the study. When there is no reaction after two weeks, the information is send again. If there is still no reaction, the contact with the legal representatives stops and the participant can not be included in the study. If an informed consent is signed, the inclusion examination of the potential participant can start. Personal information about the participant can only be inventoried after the informed consent is signed and received by the investigator.

7.3 Objection by minors or incapacitated subjects

The code 'Verzet bij wilsonbekwame (psycho) geriatrische patiënten in het kader van de Wet Medisch-Wetenschappelijk Onderzoek met Mensen' is applicable for this study. If a participant resists participating in this study, all study activities involving this participant are terminated.

7.4 Benefits and risks assessment, group relatedness

All participants, regardless of their group, receive social visits which are mostly experienced as pleasant.

There are no extra risks involved in this study. Both assessment and intervention include activities that they normally do in their daily lives. Therefore, participants are not challenged in their activities. Also, participants are supervised by well trained Master students, the professional medical help is always nearby and the participants stay in their normal everyday environment. Therefore, the risks of participating in this study are the same or even smaller than the risks of activities in normal life.

7.5 Compensation for injury

In the event that injury would occur unexpectedly, the Vu University Amsterdam offers insurance that meets the Medical Research Act. The insured amount is up to 453,780.22 Euros per participant, with a maximum of 6,806,703.24 Euros and a maximum of 9,075,604.32 euros for all the research of the institution per year.

7.6 Incentives

Subjects will receive no special incentives, compensation or treatment for participating in this study.

8. ADMINISTRATIVE ASPECTS AND PUBLICATION

8.1 Handling and storage of data and documents

All information of the participant (demographics, test results and additional medical information) will be stored under a subject code. The name of the participant can not be linked to this code since this code is not derived from initials or birth dates. When all the data is processed under the code, the name of the participant will be deleted from the coding file to guarantee privacy.

- Group (1 digit) (1= intervention, 2= control)
- Specialized nursing home (2 digits) (each specialized nursing home represents a number)
- Subject number (3 digits) (each subject is linked to a number running from 1 to 999)

The data will be treated confidentially and is only available to the coordinating researcher and principal investigator. The data is stored on a computer protected with a password. The data document on the computer is also secured by a code that only the coordinating researcher and principal investigator know. The coordinating researcher and principal investigator have access to this computer. The code connected to the names is kept by the principal investigator. Raw data will be stored in a file cabinet with a lock were only the coordinating researcher and principal investigator have access to. The key of the file cabinet is kept by the coordinating investigator.

It is unknown how long the data will be stored, depending on the follow-up after this study. If this study is a success it is possible that these data are essential for future research. If that is the case, the data are preserved for a longer period.

8.2 Public disclosure and publication policy

The results of this study will be reported in scientific articles in peer-reviewed journals. It is the responsibility of the investigator. No arrangements about publication of the study results are made with persons other than the researchers involved in this study.

REFERENCES

ACSM (1998). Position stand: the recommended quantity and quality of exercise for developing and maintaining cardio respiratory and muscular fitness, and flexibility in healthy adults. *Med Sci Sport Exerc*, 30(6):975-991.

ATS (2002). Guidelines for the six-minute walk test. *Am J Respir Crit Care Med*, 166(1):111-117.

Bossers, W (2009). Effects of physical exercise on cognition and physical functioning in older people with dementia. Master thesis Human Movement Sciences, University of Groningen.

Burns JM, Cronk BB, Anderson HS, Donnelly JE, Thomas GP, Harsha A, Brooks WM, Swerdlow RH (2008). Cardiorespiratory fitness and brain atrophy in early Alzheimer disease. *Neurology*, 71(3):210-216.

Cohen, J (1988). Statistical power analysis for the behavioral sciences (2nd ed.). Hillsdale, NJ: Lawrence Erlbaum.

Colcombe S, Kramer AF (2003). Fitness effects on the cognitive function of older adults: a meta-analytic study. *Psychol Sci*, 14(2):125-130.

Conn D, Thorpe L (2007). Assessment of behavioral and psychological symptoms associated with dementia. *Can J Neurol Sci*, 34 Suppl 1:S67-71.

Cotman CW, Berchtold NC (2002). Exercise: a behavioral intervention to enhance brain health and plasticity. *Trends Neurosci*, 25(6):295-301.

Cott CA, Dawson P, Sidani S, Wells D (2002). The effects of a walking/talking program on communication, ambulation, and functional status in residents with Alzheimer disease. *Alzheimer Dis Assoc Disord*, 16(2):81-87.

Csuka M, McCarty DJ (1985). Simple method for measurement of lower extremity muscle strength. *Am J Med*, 78: 77–81.

Ettema TP, Droes RM, de Lange J, Mellenbergh GJ, Ribbe MW (2007). QUALIDEM: Development and evaluation of a Dementia Specific Quality of Life Instrument. Scalability, reliability and internal structure. *Int J Geriatr Psychiatry*, 22: 549–556.

Ferri CP, Prince M, Brayne C (2005). Global prevalence of dementia, Alzheimer's Disease International. *The Lancet*, 366(9503):2112-2117.

Folstein MF, Folstein SE, McHugh PR (1975). Mini-Mental State: A practical method for grading the cognitive state of patients for the clinician. *J Psychiatr Res.*, 12(3):189-198.

Friedman R, Tappen RM (1991). The effect of planned walking on communication in Alzheimer’s Desease. *J Am Geriatr Soc.*, 7(39):650-659.

Johansson G, Jarnlo GB (1991). Balance training on 70-year old women. *Physioth Th Prac.*, 7:121-125.

Kempen GIJM, Miedema I, Ormel J, Molenaar M (1996). The assessment of disability with the Groningen Activity Restriction Scale. Conceptual framework and psychometric properties. *Soc Sci Med* 43:1601-1610.

Kramer AF, Hahn S, Cohen NJ, Banich MT, McAuley E, Harrison GR, Chason J, Vakil E, Bardell L, Boileau RA (1999). Ageing, fitness and neurocognitive function. *Nature*, 400:418-419.

Lautenschlager NT, Cox KL, Flicker L, Foster JK, van Bockxmeer FM, Xiao J, Greenop KR, Almeida OP (2008). Effect of physical activity on cognitive function in older adults at risk for Alzheimer disease: a randomized trial. *Am Med Assoc.*, 300(9):1077-1079.

Lexell J (2000). Muscle structure and function in chronic neurological disorders: the potential of exercise to improve activities of daily living. *Exer Sports Sci Rev*, 28(2):80-84.

Lezak MD, Howieson DB, Loring DW (2004). Neuropsychological assessment 4th edition. Oxford New York. Oxford University Press.

Lindeboom J, Jonker C (1989). Amsterdamse dementie screeningstest. Lisse, The Netherlands, Swets & Zeitlinger.

Liu-Ambrose T, Donaldson MG (2009). Exercise and cognition in older adults: is there a role for resistance training programmes? *Br J Sports Med*, 43(1):25-27.

Mannsfield Cohen et al. (1989). Nederlandse versie van de Cohen-Mansfield Agitatie Inventarisatie (CMAI-D) Cohen-Mansfield Agitation Inventory - Dutch version (CMAI-D).

Meyers BS, Alexopoulus GS (1988). Geriatric depression. *Med Clin North Am.* 72(4):847-66.

Palleschi L, Vetta F, De Gennaro E, Idone G, Sottosanti G, Gianni W, Marigliano V (1996). Effect of aerobic training on the cognitive performance of elderly patients with senile dementia of Alzheimer type. *Arch Gerontol Geriatr.*, 22(suppl. 1):47-50.

Podsiadlo D, Richardson S (1991). The timed "Up & Go": a test of basic functional mobility for frail elderly persons. *J Am Geriatr Soc.*, 39(2):142-148.

Robey, RR (2004). Reporting point and interval estimates of effect-size for planned contrasts: fixed within effect analyses of variance. *J Fluency Disord.*, 29(4): 307-341.

Rossiter-Fornoff JE, Wolf SL, Wolfson LI, Buchner DM (1995). A cross-sectional validation study of the FICSIT common data base static balance measures. Frailty and Injuries: Cooperative Studies of Intervention Techniques. *J Gerontol A Biol Sci Med Sci,* 50(6):291-297.

Salloway S, Correia S: Alzheimer disease (2009). Time to improve its diagnosis and treatment. *Cleve Clinic J Med.*, 76(1):49-58.

Scarborough DM, Krebs DE, Harris BA (1999). Quadriceps muscle strength and dynamic stability in elderly persons. *Gait & posture*, 10(1):10-20.

Schafer JH, Glass TA, Bolla KI, Mintz M, Jedlicka AE, Schwartz BS (2005). Homocysteine and cognitive function in a population-based study of older adults. *J Am Geriatr Soc*, 53(3):381-388.

Scherder E, Eggermont L, Sergeant J, Boersma F (2007). Physical activity and cognition in Alzheimer's disease: relationship to vascular risk factors, executive functions and gait. *Rev neurosci.*, 18(2):149-158.

Snijders JT (1983). Groninger Intelligentie Test. Lisse: Swets & Zeitlinger.

Sobel BP (2001). Bingo vs. physical intervention in stimulating short-term cognition in Alzheimer's disease patients. *Am J Alzheimetrs Dis Other Dem.*, 16(2):115-120.

Steffen T.M, Hacker T.A, Mollinger L (2002). Age- and gender-related test performance in community-dwelling elderly people: six-minute walk test, berg balance scale, timed up & go test, and gait speeds. *Physical Therapy.* 82(2):128-37.

Suurmeijer TBPM Doeglas DM et al. The Groningen Activity Restriction Scale for measuring disability: Its utility in international comparisons. *Am J Public Health.* 84: 1270-1273.

Tappen RM, Roach KE, Buchner D, Barry C, Edelstein J (1997). Reliability of physical performance measures in nursing home residents with Alzheimer's disease. *J Gerontol A Biol Sci Med Sci*, 52(1):M52-55.

Tegner Y, Lysholm J, Lysholm M, Gillquist J (1986). Strengthening exercises for old cruciate ligament tears. *Acta Orthop Scand*, 57(2):130-134.

Thomas VS, Hageman PA (2002). A preliminary study on the reliability of physical performance measures in older day care center clients with dementia. *Int Psychogeriatr.*, 14(1):17-23.

Thomas VS, Hageman PA (2003). Can neuromuscular strength and function in people with dementia be rehabilitated using resistance-exercise training? Results from a preliminary intervention study. *J Gerontol A Biol Sci Med Sci*, 58(8):746-751.

Tiedemann A, Shimada H, Sherrington C, Murray S, Lord S (2008). The comparative ability of eight functional mobility tests for predicting falls in community-dwelling older people. *Age and Ageing*, 37: 430–435.

Van Heuvelen MJG, Kempen GIJM, Brouwer WH, de Greef MHG (2000). Physical fitness related to disability in older persons. *Gerontology*, 46: 333-341.

Verkerke GJ, Lemmink KA, Slagers AJ, Westhoff MH, van Riet GA, Rakhorst G (2003). Precision, comfort and mechanical performance of the Quadriso-tester, a quadriceps force measuring device. *Med Biol Eng Comput*, 41(3):283-289.

Wechsler D (1987). Manual for the Wechsler Memory Scale-Revised. San Antonio, The Psychological Corporation.

Wechsler D (1997). WAIS-III: Wechsler Adult Intelligence Scale, Administration and Scoring Manual. 3rd ed. edition. New York, Psychological Corporation.

Wilson BA, Cockburn J, Baddeley AD (1985). The Rivermead Behavioural Memory Test Manual. Bury, Valley Test Co.

Zuidema SU, de Jonghe JF, Verhey FR, Koopmans RT (2007). Agitation in Dutch institutionalized patients with dementia: factor analysis of the Dutch version of the Cohen-Mansfield Agitation Inventory. *Dement Geriatr Cogn Disord.* 23(1):35-41.
